# Diffusion tensor imaging and electrophysiology as robust assays to evaluate the severity of acute spinal cord injury in rats

**DOI:** 10.1186/s12883-020-01778-1

**Published:** 2020-06-09

**Authors:** Beike Chen, Qiang Tan, Weikang Zhao, Qiming Yang, Hongyan Zhang, Fabao Gao, Xin Liu, Hua Feng, Dianming Jiang

**Affiliations:** 1 Department of Orthopedics, The First Affiliated Hospital of Chongqing Medical University, Chongqing, 400016 People’s Republic of China; 2 Department of Neurosurgery, Southwest Hospital, Third Military Medical University, Army Medical University, Chongqing, 400038 People’s Republic of China; 3 Department of Radiology, West China Hospital, Sichuan University, Chengdu, 610041 Sichuan People’s Republic of China; 4 Department of Orthopedics, The Third Affiliated Hospital of Chongqing Medical University (Gener Hospital), Chongqing, 401120 People’s Republic of China

**Keywords:** Anisotropy, DTI, SCI, MEPs, BBB, White matter, Neuroimaging

## Abstract

**Background:**

Diffusion tensor imaging (DTI) is an effective method to identify subtle changes to normal-appearing white matter (WM). Here we analyzed the DTI data with other examinations, including motor evoked potentials (MEPs), histopathological images, and behavioral results, to reflect the lesion development in different degrees of spinal cord injury (SCI) in acute and subacute stages.

**Method:**

Except for 2 Sprague -Dawley rats which died from the anesthesia accident, the rest 42 female rats were randomized into 3 groups: control group (*n* = 6), moderate group (*n* = 18), and severe group (*n* = 18). Moderate (a 50-g aneurysm clip with 0.4-mm thickness spacer) or severe (a 50-g aneurysm clip with no spacer) contusion SCI at T8 vertebrae was induced. Then the electrophysiological assessments via MEPs, behavioral deterioration via the Basso, Beattie, and Bresnaha (BBB) scores, DTI data, and histopathology examination were analyzed.

**Results:**

In this study, we found that the damage of WM myelin, MEPs amplitude, BBB scores and the decreases in the values of fractional anisotropy (FA) and axial diffusivity (AD) were more obvious in the severe injury group than those of the moderate group. Additionally, the FA and AD values could identify the extent of SCI in subacute and early acute SCI respectively, which was reflected in a robust correlations with MEPs and BBB scores. While the values of radial diffusivity (RD) showed no significant changes.

**Conclusions:**

Our data confirmed that DTI was a valuable in ex vivo imaging tool to identify damaged white matter tracts after graded SCI in rat, which may provide useful information for the early identification of the severity of SCI.

## Background

Early intervention in acute stage of spinal cord injury (SCI) can reduce the adverse effect of glial scar and cavity on axon regeneration, showing a superiority in spinal cord repair [1]. Timely and effective identification of SCI severity, which provides important information and therefore affects early clinical decision-making, is the key to early intervention [2, 3]. The identification of the severity of SCI commonly depends on the International Standards for the Neurological Classification of Spinal Cord Injury [4, 5]. However, since the neurological responses can be affected by the spinal shock in acute stage of SCI, the reliability of current standards, which is based on the strength of various muscles as well as neurological responses to sensory input on both sides of the body, for early identification of the severity of SCI is limited [6].

Currently, magnetic resonance imaging (MRI) is the main imaging modality to visualize the injury characteristics of SCI [7]. However, since the integrity of white matter tract is the main factor affecting the function outcome of SCI patients, and conventional T2 weighted images is not suitable for the evaluation of white matter tracts, the utility of anatomic MRI as a prognostic tool is limited during the acute phase of SCI [8]. Diffusion tensor imaging (DTI), weighted with the local microstructural characteristics of water diffusion, provides an effective means of visualizing functional connectivity in the nervous system, and is being used to identify subtle changes to the normal-appearing white matter on anatomic MRI [9–11]. As a noninvasive method, DTI also showed superiority on differentiation of low-grade from high-grade gliomas and residual/recurrent gliomas from post-radiation changes [12, 13]. Although the changes of spinal cord after acute injury has been observed by DTI, the relationship between these changes and the degree of injury is unclear.

Electrophysiology, such as motor evoked potentials (MEPs), is a good method to evaluate the functional integrity of spinal pathways [14], and has been used to identify connected but nonfunctional axons crossing SCI lesions in rodents [15], dogs [16], and non-human primates [17]. The MEPs amplitude in rat SCI model has been reported to be positively correlated with the preserved tissue at the injured site [18, 19]. The permeability of the axonal membrane to water is intimately tied to MR diffusion measurements [20], as well as the morphometry tied to neuronal excitability [21]. Therefore, it is reasonable to assume that the changes in DTI measurements is correlated with the MEP parameters.

Accordingly, the consistency and capability of DTI to reflect the lesion development, as well as the relationship between imaging and other examinations in different degrees of SCI in acute stage, was explored in a most commonly used rat model.

## Methods

### Ethic oversight

Animal experimental procedures were performed under the Regulations for the Administration of Affairs Concerning Experimental Animals approved by the State Council of People’s Republic of China. All experiments were conducted in accordance with animal care guidelines approved by the Laboratory Animal Welfare and Ethics Committee of Third Military Medical University (the Army Military Medical University).

### Study design and subjects

All experiments were performed in the central laboratory in Southwest Hospital except for the ex vivo DTI acquisition and analyses, which were performed in the laboratory of department of Molecular Imaging in West China Hospital.

Adult female Sprague-Dawley (SD) rats (aged approximately three-months-old, 200-250 g) were obtained from the Army Military Medical University. Animals were housed in a controlled pathogen-free environment at a temperature of 23 ~ 25 °C, 70% humidity, 12 h light–dark cycles, free access to food and water.

The sample size was calculated according to the previous study [22]. Except for 2 rats died from anesthesia accident, the rest 42 rats were randomized into 3 groups: control group (*n* = 6), moderate group (*n* = 18), and severe group (*n* = 18). Six rats from each group were tested for Basso, Beattie, and Bresnaha (BBB) score and MEPs test at different timepoints to evaluate the graded SCI injury in rats, and then animals were sacrificed on day 14 for ex vivo DTI and histological examination to provide imaging evidences. The rest animals from both moderate and severe group were sacrificed on day 3 /7 (*n* = 6/group at each timepoint) for ex vivo DTI and histological examination. The euthanasia was performed with an intraperitoneal injection of 1% sodium pentobarbital overdoses (100 mg/kg body weight).

### SCI model

SCI was induced according to the previous method [23, 24]. Briefly, rats were anesthetized with 1% sodium pentobarbital (40 mg/kg body weight, i. p.), and fixed in a prone position to perform a dorsal laminectomy at the T7-9vertebrae. A 50-g aneurysm clip was used to laterally compress the T8 spinal cord with no spacer for 30 s to establish the severe injury model, while adding a 0.4-mm thickness spacer to establish the moderate injury model. The control group received identical laminectomy without crush injury. The skin incision was then closed. SCI rats were isolated to a separate cage in the animal house. Antibiotics (Cefazolin, 50 mg/kg body weight, i. p.) were given daily to treat/prevent bladder infection immediately after the surgery for 7 continuous days. Bladders were pressed twice daily until the rats recovered to normal spontaneous micturition.

### Behavioral assessment of motor function

All animals were assessed in an open-field walking according to the BBB locomotor scale [25], the day before inducing injury, and on day 1/3/7/14 after operation. Hindlimb function was assessed according to the 0–21 BBB scoring, where 0 is flaccid paralysis and 21 is normal gait.

### Electrophysiological assessments of hindlimb motor function

MEPs were induced according to the previous method [26, 27]. Briefly, rats were anesthetized with 1% pentobarbital sodium (20 mg/kg i.p.). The stimulation needle electrode (DSN1620, Medtronic, USA), acting as the anode, was inserted subcutaneously at the base of the nose with the tip point touching the scalp. The needle electrode acting as the cathode was placed subcutaneously at the midpoint of the two ears. The recorded electrode was inserted into the tibialis anterior (TA) muscle. A ground electrode was placed subcutaneously at the base of the tail. A single pulse of electrical stimulation (10 mA, 0.1 ms, 1 Hz) to excite the brain was delivered via stimulator (Keypoint, Medtronic, USA). The electrical stimulation was repeated five times at an interval of 15 s in each rat. The base-to-peak amplitude of the trace after single stimulation was recorded for each type of evoked potential.

### Histology

Rats were sacrificed at 3 days (3d), 7 days (7d) and 14 days (14d) post SCI. The spinal cords were harvested, fixed, dehydrated, embedded, and sliced into 20 μm successively. After blocked with 5% bovine serum albumin (BSA) for 1.5 h at room temperature, the slices were incubated with the primary antibodies of goat anti-myelin basic protein (anti-MBP; 1:250; Santa Cruz; Cat: sc-13,914) overnight at 4 °C, and were probed subsequently with AlexaFluor-488-conjugated secondary antibodies against goat (1:200; Invitrogen) antibodies for 2 h at 37 °C. The nuclei were counterstained with 4^′^ -6-diamidino-2-phenylindole (DAPI; Santa Cruz Biotechnology). Sections were imaged under a Zeiss confocal microscope (Zeiss, LSM780).

### Ex vivo DTI acquisition

Magnetic resonance imaging was performed with a 7.0 Tesla Bruker Biospec 70/30 USR preclinical scaner, using a volume coil with a 23-mm inner diameter and 44-mm outer diameter for both transmission and reception. The spinal cord specimens were loaded into 2 mL syringes filed with Fomblin (Solvay, Brussels, Belgium) (28) and stabilized by a parallel plastic rod (6-mm diameter). RARE T2-weighted scans were acquired to locate the epicenter of the injury. Fifteen thick slices arranged around the epicenter were acquired for diffusion tensor imaging (sequence parameters: spin echo DTI-EPI, b = 1319s/mm^2^, δ = 4 ms, Δ = 18 ms, 30 diffusion sampling directions, 16 averages, TE/TR = 32/2750 ms, 100 μm in-plane resolution, acquisition time = 3h6min40s).

Paravision Version 5.0 (Bruker BioSpin) was used for the analyses of B0, fractional anisotropy (FA) and mean diffusivity (MD) map. The cross-sectional areas of the spinal cord were defined by manually outlining the white matter (WM) areas on transverse B0 and FA scans for each time point. Of the 15 diffusion axial images acquired, the 11 axial slices centered on the lesion core were used for subsequent. Regions of interest (ROIs), which chose the total WM were manually outlined on the transverse images.

The DTI metrics including λ1, λ2, λ3, MD, and FA were used with the Paravision Version 5.0 (Bruker BioSpin, Karlsruhe, Germany) diffusion tensor calculation module according to the previous study [28]. The axial diffusivity (AD) is a measure of the diffusivity along the principal axis of the diffusion tensor (AD = λ1). The radial diffusivity (RD) was calculated using the two minor diffusion axes (RD = (λ2 + λ3) / 2). The FA was calculated using the equation: FA = √1/2(√((λ1 − λ2)^2^ + (λ1 − λ3)^2^ + (λ2 − λ3)^2^) / √(λ1^2^ + λ2^2^ + λ3^2^)).

Fibertracks were visualised and analyzed using TrackVis (v. 0.6.1) by drawing ROIs on epicentral mask in ex vivo B0 maps for ROI filers. Then count the number of tracks which were greater than 3 mm was counted.

### Statistical analysis

All DTI metrics were presented as mean and standard error of the mean (SEM). BBB score, MEPs amplitude and number of tracks were presented as mean and standard deviation (SD). BBB score, MEPs and DTI metrics data were analyzed using one-way analysis of variance (ANOVA), and group differences were ascertained using Tukey’s post hoc comparisons when appropriate. Comparisons between 2 groups were analyzed using 2-tailed Student t-tests. DTI metrics (including FA, AD and RD) of the epicentral spinal cord were analyzed relatively to BBB and MEPs via Pearson’s correlation. All statistical analyses were performed via the SPSS 23.0 software. The significance level for all tests were set at *P* < 0.05.

## Result

### Graded SCI led to changes of locomotor capacity and MEPs amplitude

**Fig. 1:**
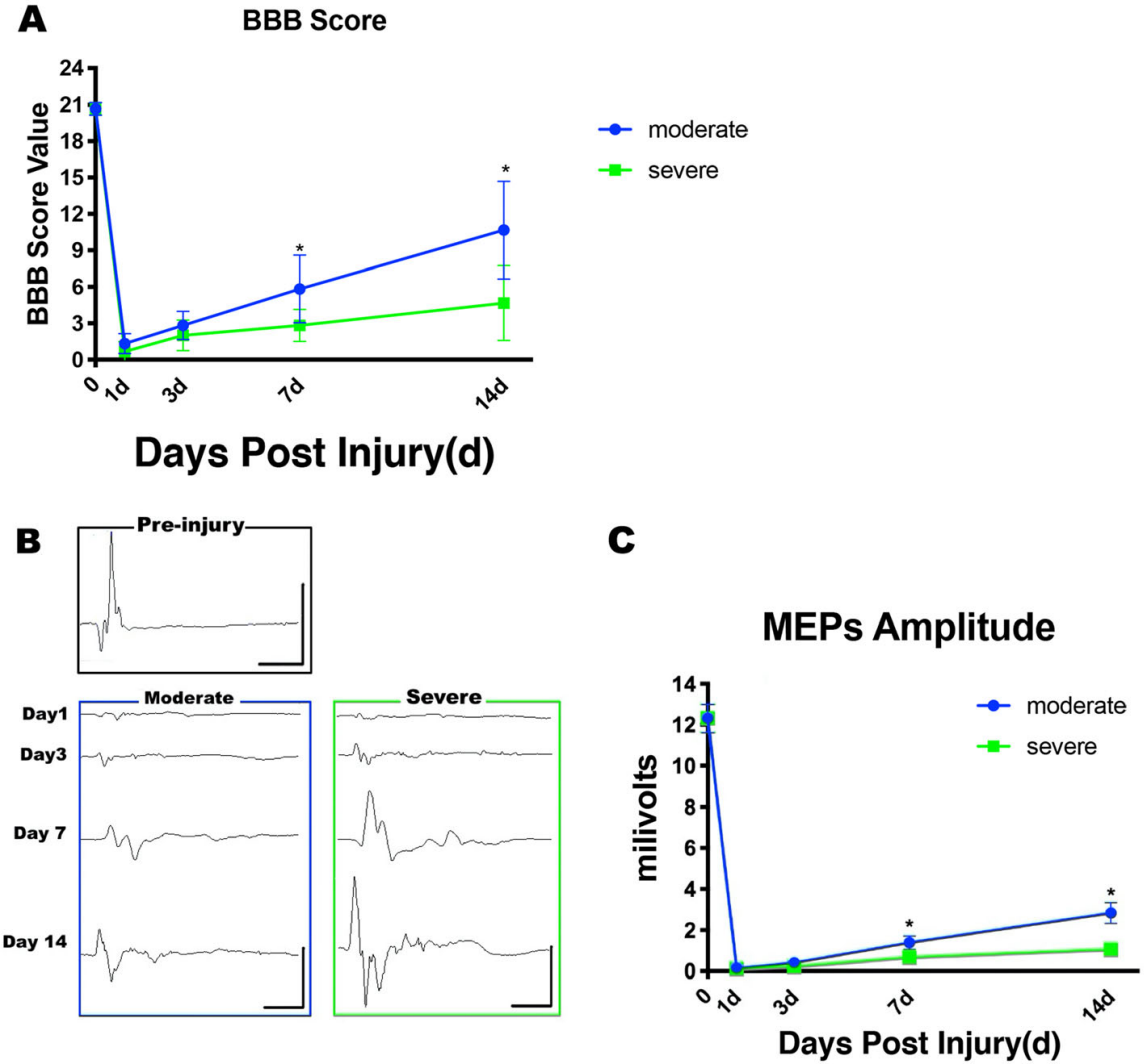
Hindlimb motor function after different severities of SCI. **a** The BBB scores was similar between the moderate group and severe group on day 1 to day 3, but reached a significant difference from day 7 to day 14 (6 rats/group). **b** Representative recording of MEPs from control group, moderate and severe groups at various time points. **c** The MEPs was similar between the moderate group and severe group on day 1 to day 3, but reached a significant difference from day 7 to day 14 (6 rats/group). Results are presented as mean ± SD, ^∗^*P* < 0.05. Scale: 5 mV/10 ms in pre-injury recordings, and 2 mV/10 ms at other time points

All rats were tested for locomotor capacity using the BBB locomotor scale. On day 1, both moderate and severe group showed nearly complete paralysis, which was reflected in a BBB score of 0 ~ 2 (Fig. 1a). Then the animal’s locomotor capacity began to recover gradually. On day 7 and 14, the BBB scores of the moderate group were both higher than those of the severe group (*P* < 0.05), which indicated a better functional recovery in the moderate group. After the first day of SCI, both moderate and severe group exhibited a markedly reduced amplitude in MEPs. Then, the amplitude of MEPs rebounded gradually to that in second week (Fig. 1b). On day 7 and 14, the severe group had a lower MEPs amplitude than the moderate group (moderate vs severe, *P* < 0.05, Fig. 1b and c), which suggested that the abnormal MEPs, as well as the central conduction, can still transmit through the spinal cord in relation to the severity of the injury.

### Graded SCI was reflected by DTI-based morphometrics and metrics from the ventrolateral white matter (VWM) area

**Fig. 2:**
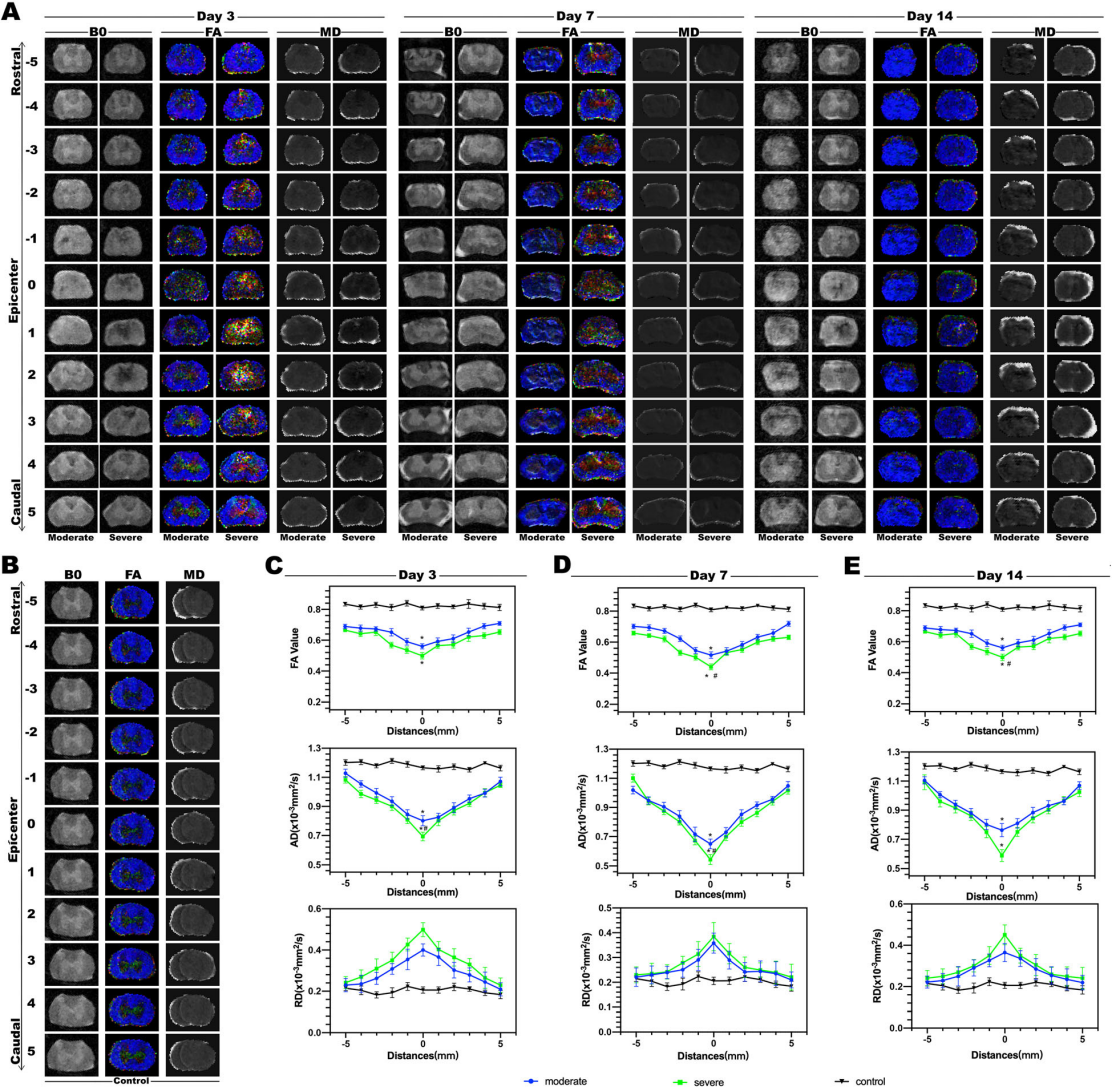
Alterations in Diffusion Tensor Imaging (DTI) after different severities of SCI. **a** Representative DTI images after SCI, including B0 images, FA colour map and MD map from rostral to caudal of white matters. **b** Representative DTI images from control group. **c**-**e** Mean values of FA, AD and RD at the VWM of epicenter were analyzed. The values of FA and AD decreased after SCI (6 rats/group, * *P* < 0.05), and the severe group showed more significant decline in the FA values on day 7 and 14, so do the AD values on day 3 and 7. The RD value showed no significant changes after SCI

The variations of the DTI metrics from the VWM area and typical B0, FA and MD maps from the epicenter towards the rostral and caudal were showed in Fig. 2. The values of FA and AD at the epicenter decreased significantly after SCI (moderate, severe vs control, *P* < 0.05). Compared with the moderate group, the severe group exhibited a more significant decline in FA value and AD value on day 7/14 and 3/7 respectively (moderate vs severe, *P* < 0.05). The RD value in different positions of both moderate and severe groups were distributed in peak shape, and the value in the epicenter at all time points were similar (*P* > 0.05).

### Graded SCI on diffusion tensor tractography

**Fig. 3:**
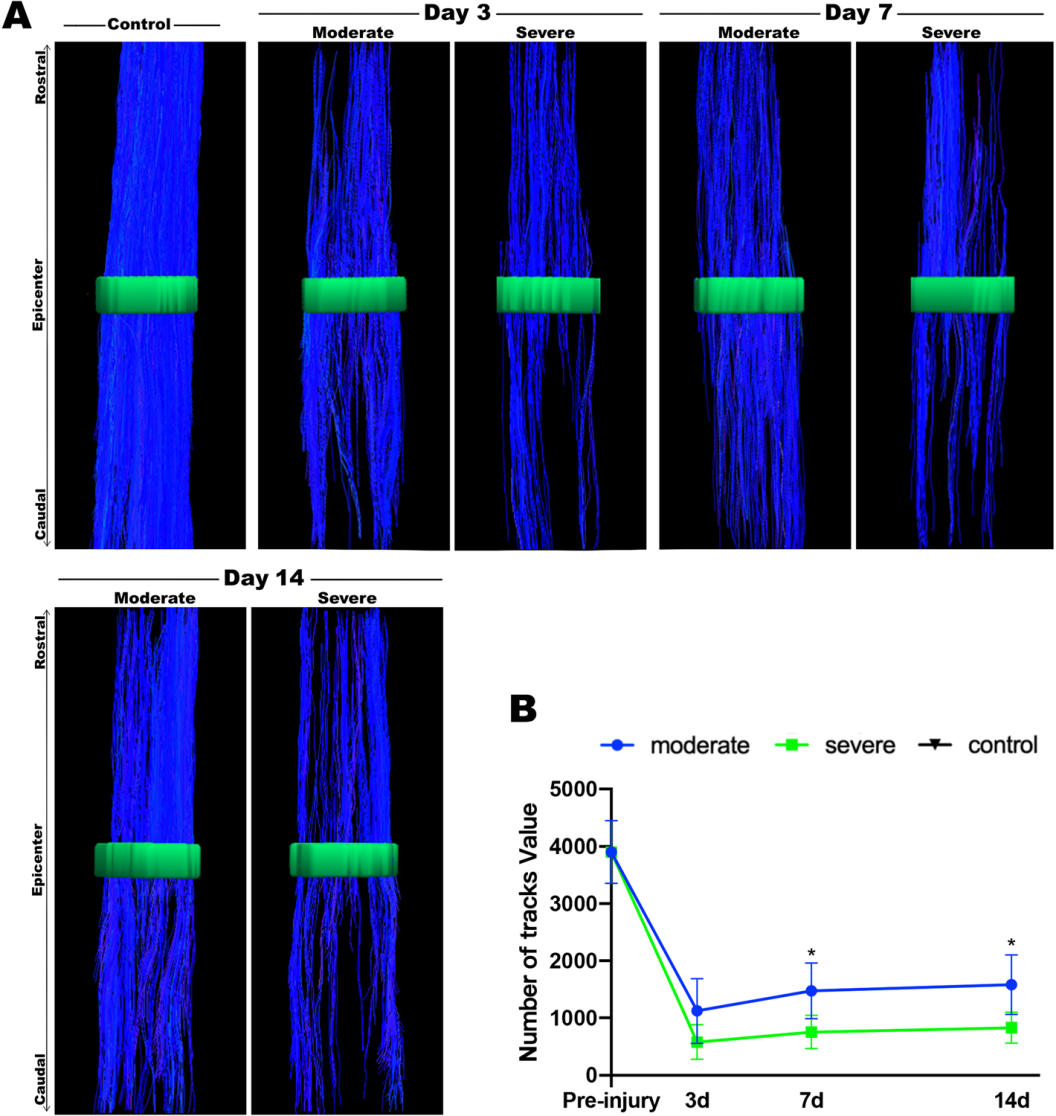
White matters tracks detected by DTT (diffusion tensor tractography) after different severities of SCI. **a** Representative DTT images. The green ROIs were the epicenter of white matters. White matters fibers were represented by blue lines. **b** Quantitation of white matters tracks across the ROIs. Results are presented as mean ± SD, ^∗^*P* < 0.05

Based on the rat in ex vivo diffusion tensor data, we created fibertracks to depict the diffusion tensor tract fibers (DTT) of the white matter through the epicenter. In the DTT, each path was described in blue colour according to its superior-inferior (rostral-caudal) diffusion direction. In the white matter of normal spinal cord, we observed compact and orderly blue fiber tracts (Fig. 3a). The number of tracks across the epicenter of the white matters in the moderate groups was more than that in the severe groups (Fig. 3b) on day 7 and 14 (*P* < 0.05), but similar on day 3 (*P* > 0.05).

### The effect of graded SCI on the integrity of white matter myelin

**Fig. 4:**
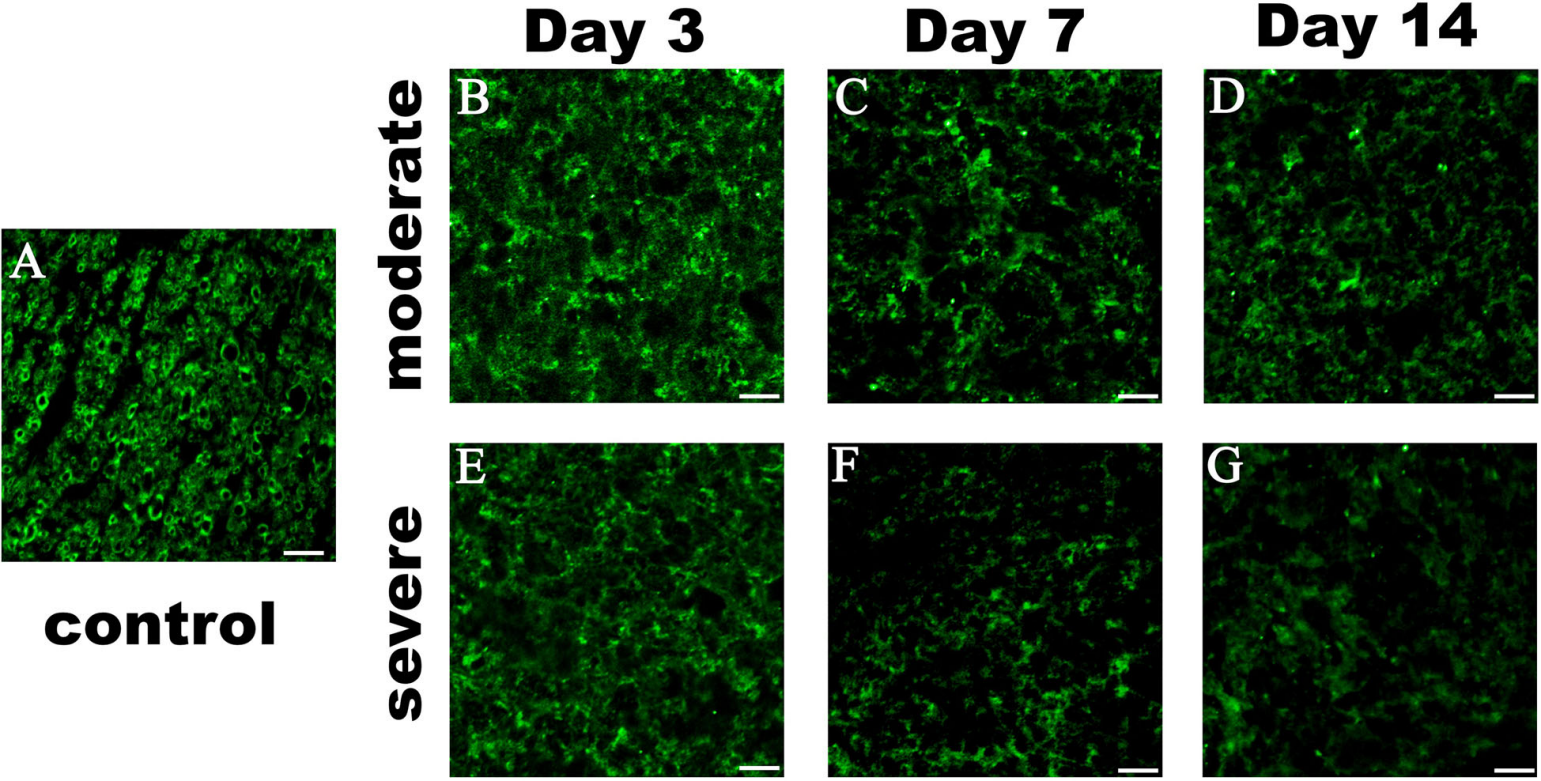
Myelin changes after different severities of SCI. **a** Representative images of immunofluorescence staining for myelin (MBP, green) in theVWM area. **b-c** After moderate SCI, myelin is disrupted and contains patches of reduces of reduced staining and/or loss of the characteristic of green rings, as well as in severe group (**e-f**) but more highly disrupted. Scale bars represent 40 μm

In the control group, Myelin Basic Protein (MBP) immunofluorescence was visualized as the characteristic ring in the VWM area. After injury, complete rings of myelin could not be seen at the same areas of epicenter (Fig. 4a-g), which suggested that the MBP fluorescence intensity decreased and myelin was markedly deteriorated in relation to the severity of injury.

### Correlation between DTI metrics and locomotor capacity /MEPs amplitude after SCI

**Fig. 5:**
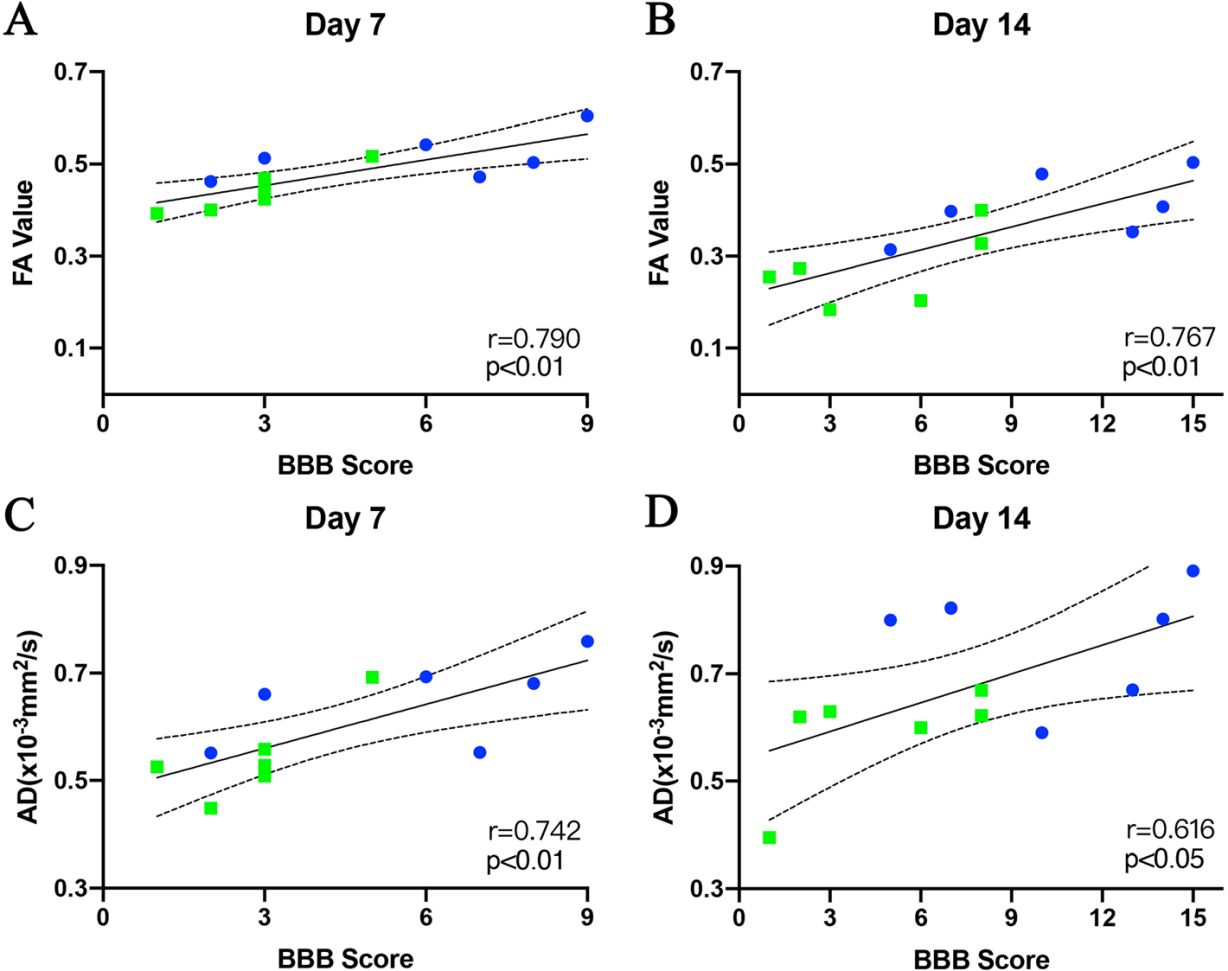
Correlation analyses of the values of FA and AD with BBB Scores. FA and AD value were significantly correlated with BBB scores on day 7(**a**, **c**) and 14 (**b**, **d**). Blue dots represented rats with moderate SCI and green squares represented those with severe SCI. Solid line shows line of best fit; dotted lines shows 95% confidence interval

**Fig. 6:**
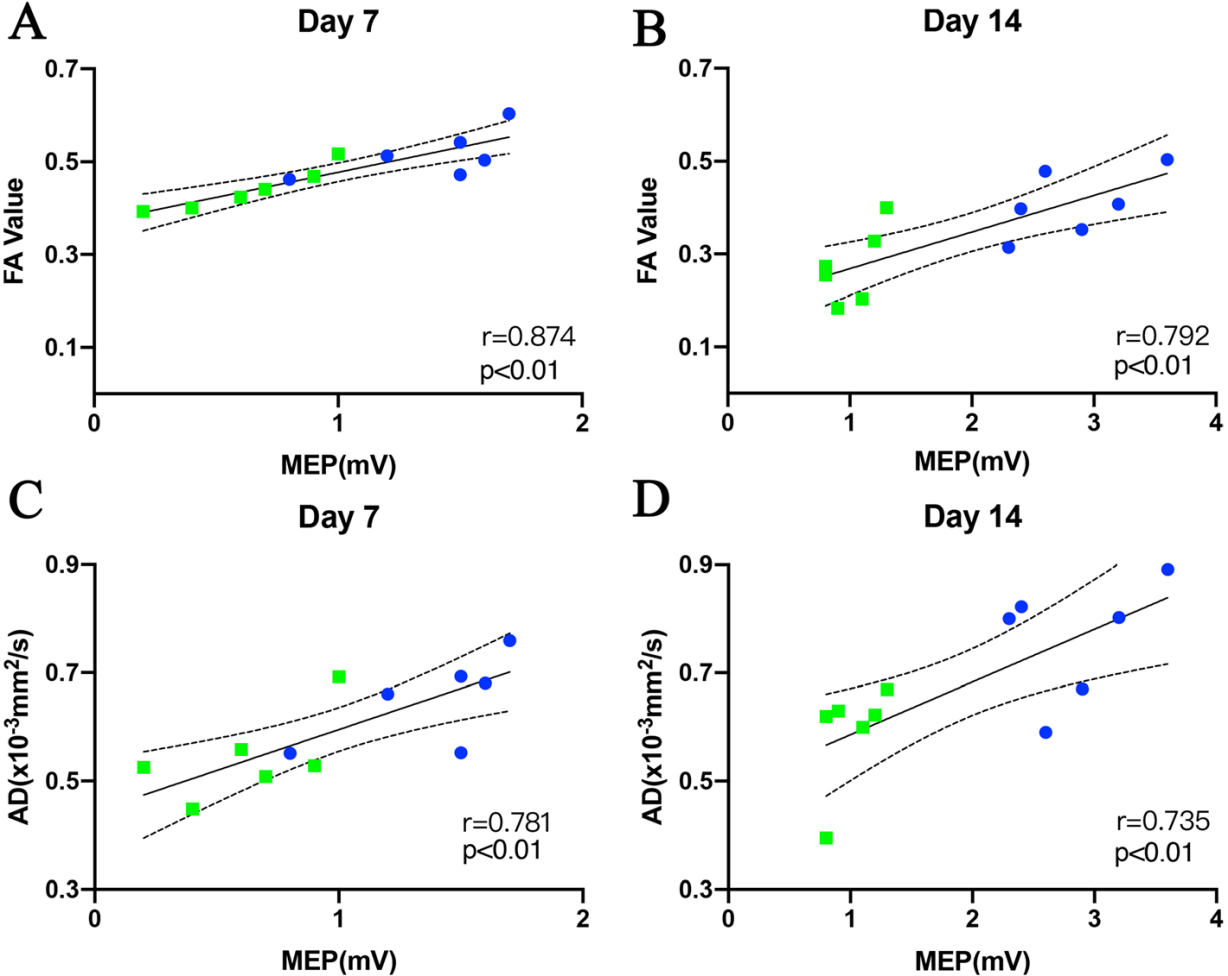
Correlation analyses of the values of FA and AD with MEPs. FA and AD value were significantly correlated with MEPs on day 7(**a**, **c**) and 14 (**b**, **d**). Blue dot represented rats with moderate SCI and green square represented those with severe SCI. Solid line shows line of best fit; dotted lines shows 95% confidence interval

Correlation analyses demonstrated that the BBB scores were significantly correlated with epicentral FA values of the VWM area at day 7 (*r* = 0.790; *P* < 0.01) and 14 (*r* = 0.767; *P* < 0.01; Fig. 5a, b), as well as the epicentral AD values of the VWM area at day 7 (*r* = 0.741; *P* < 0.01) and 14 (*r* = 0.616; *P* < 0.05; Fig. 5c, d). Similarly, MEPs were significantly correlated with epicentral FA values of the VWM area at day 7 (*r* = 0.874; *P* < 0.01) and 14 (*r* = 0.792; *P* < 0.01; Fig. 6a, b), as well as, epicentral AD values of the VWM area at day 7 (*r* = 0.781; *P* < 0.01) and 14 (*r* = 0.735; *P* < 0.01; Fig. 6c, d). This shows the capacity of FA and AD values to accurately assess motor function.

## Discussion

In this study, we used ex vivo diffusion tensor imaging to assess the graded severities of contusion SCI at different time points and analyzed the correlation between imaging results and corresponding electrophysiological and behavioral outcome. The results suggested that DTI might be an important noninvasive technique to diagnose the severity of acute and subacute SCI, as well as a tool to evaluate functional changes. The AD and FA values were the most robust diffusion MRI indicator of damage in early acute and subacute SCI, respectively. Additionally, FA in VWM area showed a most robust correlation with MEP and BBB score at day 7. To our best knowledge, it is the first time that the correlation between the MEP and DTI metrics after acute SCI has been explored.

The conventional MRI, like T2WI, is regard as a useful modality for the visual assessment of spinal cord [29]. However, it is not suitable for identifying the integrity of WM tracts in acute SCI [8]. Diffusion-weighted MR imaging was sensitive to identify the structural changes or/and edema grade differences in human diseases, like gliomas [12, 13], paraspinal neurogenic tumor [30], pathologic fracture [31] and SCI [32]. By correlating the diffusion-weighted MR imaging metrics with biopsy results of the mediastinal neurogenic tumor, a retrospective analysis found that the ADC values could be used to differentiate malignant from benign neurogenic tumors and schwannomas from neurofibromas with a high accuracy and sensitivity [30]. Different diffusion MRI metrics mean different in SCI: reduced FA indicates the demyelination of spared axon and axonal damage, as well as decreased AD means axonal collapse [33, 34], while RD changes may correlate with the damage across the myelin sheath [35, 36]. In the present study, we assessed the sequential FA, AD and RD values at the VWM of the rostro-caudal sector and found that the FA and AD values in the severe group were the lowest at the lesion epicenter at every time points. The FA and AD values did not always change in parallel. FA did not show any significant changes over time in both two severities of acute SCI. However, AD showed significant changes between day 3 and 7. This characteristic might be due to the detectable axonal collapse at the early time points of acute SCI, which was consistent with previous research results [37, 38]. The RD values of the lesion epicenter in the severe group remained the highest but did not change with time and severities. Since the different parameters reflected different problems, it was necessary to combine multiple metrics for comprehensive analysis. A clinic study found that combination of DTI metrics could be used for differentiating malignant from benign compressed vertebrae as the malignant revealed lower MD and higher FA than benign involvement [31]. As to our work, the FA, AD, and RD values changed differently, so we needed to combine more detection methods to find out which value could reflect the damage of graded SCI over time more accurately.

MEP testing, which is regarded as an objective measure to quantifiably evaluate functional integrity of spinal pathways [39], is also a sensitive tool to detect the changes in conductivity of spinal cord. Because the principle of DTI and MEP measurements are related to the morphometry and permeability of the axonal membrane to water [20, 21], we analyzed the correlation between the two methods, and found a parallel alteration in both MEP amplitudes and DTI measures (FA and AD). What’s more, the current investigation showed significant correlations between acute (day 7 and 14) DTI metrics and acute BBB scores, as well as histopathology, which reflected the previous study in adults and rats [40, 41]. Based on the above results, we concluded that AD value could accurately reflect the degree of spinal cord injury in the early acute phase, while FA seemed to show superiority in the subacute phase.

There are several limitations in our study. First, we preformed ex vivo in order to ensure high SNR and avoid the artifacts which would augment in vivo, but it is impossible to perform MRI scan on the same subject over time, and we will probably miss some details of the injury processes which will be obvious in vivo. In fact, although several studies have applied DTI to the acute SCI both in animal models as well as human subjects [11], limited by the significant artifact from respiratory and cerebrospinal fluid (CSF) motion, it still has difficulties in imaging and evaluation of the spinal cord [42], which needs further studies. We are going to find normative values of DTI metrics in vivo. Second, because of the injury, it is difficult to delineate the boundary of WM/GM, which will cause more variability in DTI metrics.

## Conclusions

Using behavior evaluation, electrophysiological assessments, DTI and morphology, the present study examined the dynamic variation of acute SCI and explored the correlation between MEP and motor function in rats. It is very important to understand the pathophysiology and functional changes of acute SCI. Through such assessment, we may provide a useful information for the early identification of the severity of SCI and therefore give an optimal treatment.

## Data Availability

The datasets used and/or analysed during the current study are available from the corresponding author on reasonable request.

## Abbreviations

DTI: Diffusion tensor imaging
WM: White matter
SCI: Spinal cord injury
MEPs: Motor evoked potentials
BBB: Basso: Beattie: and Bresnaha
FA: Fractional anisotropy
AD: Axial diffusivity
RD: Radial diffusivity
MRI: Magnetic resonance imaging
SD: Sprague-Dawley
BSA: Bovine serum albumin
ROIs: Regions of interest
MD: Mean Diffusivity
SEM: Standard error of the mean
SD: Standard deviation
ANOVA: Analysis of variance
VWM: Ventrolateral white matter
DTT: Diffusion tensor tract fibers
MBP: Myelin Basic Protein
DPI: Days postinjury
